# The low affinity glucose transporter HxtB is also involved in glucose signalling and metabolism in *Aspergillus nidulans*

**DOI:** 10.1038/srep45073

**Published:** 2017-03-31

**Authors:** Thaila Fernanda dos Reis, Benjamin M. Nitsche, Pollyne Borborema Almeida de Lima, Leandro José de Assis, Laura Mellado, Steven D. Harris, Vera Meyer, Renato A. Corrêa dos Santos, Diego M. Riaño-Pachón, Laure Nicolas Annick Ries, Gustavo H. Goldman

**Affiliations:** 1 Faculdade de Ciências Farmacêuticas de Ribeirão Preto, Universidade de São Paulo, Ribeirão Preto, São Paulo Brazil; 2 Applied and Molecular Microbiology, Institute of Biotechnology, Berlin University of Technology, Gustav-Meyer-Allee 25, 13355, Berlin, Germany; 3 Center for Plant Science Innovation and Department of Plant Pathology, University of Nebraska, Lincoln, Nebraska 68588-0660, United States of America; 4 Laboratório Nacional de Ciência e Tecnologia do Bioetanol (CTBE), Centro Nacional de Pesquisa em Energia e Materiais (CNPEM), Campinas, São Paulo, Brazil

## Abstract

One of the drawbacks during second-generation biofuel production from plant lignocellulosic biomass is the accumulation of glucose, the preferred carbon source of microorganisms, which causes the repression of hydrolytic enzyme secretion by industrially relevant filamentous fungi. Glucose sensing, subsequent transport and cellular signalling pathways have been barely elucidated in these organisms. This study therefore characterized the transcriptional response of the filamentous fungus *Aspergillus nidulans* to the presence of high and low glucose concentrations under continuous chemostat cultivation with the aim to identify novel factors involved in glucose sensing and signalling. Several transcription factor- and transporter-encoding genes were identified as being differentially regulated, including the previously characterized glucose and xylose transporter HxtB. HxtB was confirmed to be a low affinity glucose transporter, localizing to the plasma membrane under low- and high-glucose conditions. Furthermore, HxtB was shown to be involved in conidiation-related processes and may play a role in downstream glucose signalling. A gene predicted to encode the protein kinase PskA was also identified as being important for glucose metabolism. This study identified several proteins with predicted roles in glucose metabolic processes and provides a foundation for further investigation into the response of biotechnologically important filamentous fungi to glucose.

Second generation (2G) biofuel production from lignocellulose-rich plant biomass has gained considerable interest as a renewable, environmentally friendly and food crop non-competitive energy source^1^. Biofuel production from lignocellulose encompasses 4 steps: (i) pre-treatment of the raw lignocellulosic material, (ii) enzymatic hydrolysis and release of simple sugars, (iii) fermentation of these sugars and (iv) purification and distillation of the biofuels^2^. Enzymatic hydrolysis of lignocellulosic polysaccharides and subsequent fermentation are of great research interest in order to make 2G biofuel production a cost-effective process.

Filamentous saprotrophic fungi, such as *Aspergillus spp*. and *Trichoderma reesei*, which are used in various industrial applications, are currently being investigated for the improved production of enzymes that hydrolyse plant sugar polymers^3^. Lignocellulose is composed of a wide range of different polysaccharides and saprotrophic fungi secrete a diverse array of enzymes that can degrade complex lignocellulose saccharides into simpler, fermentable sugars^4^,^5^. One of the drawbacks of this procedure is the inhibition of enzyme secretion in the presence of glucose, a hexose sugar which constitutes the basic unit of cellulose; a polymer which makes up 40–60% of the plant cell wall^4^,^5^.

Glucose is the preferred carbon source for most microorganisms as it is easily metabolized and provides quick energy for growth and niche colonization^6^. In *Aspergillus spp*. and *T. reesei*, the detection of glucose triggers the repression of genes encoding enzymes required for lignocellulose degradation, a mechanism known as carbon catabolite repression (CCR) and mediated by the transcription factor CreA/CRE1. Although CreA/CRE1-mediated transcriptional repression has been extensively studied^6^,^7^,^8^,^9^,^10^, sensing of extracellular glucose concentrations and subsequent transport have been barely characterized in filamentous fungi.

In *S. cerevisiae*, Rgt2p and Snf3p are two sensors that upon glucose detection increase the expression of transporters, therefore enhancing glucose uptake^11^. In the absence of glucose, Rgt1p forms a repressor complex with Mth1p, Std1p and Ssn6p-Tup1p, inhibiting the expression of *HXT* transporter-encoding genes^11^. Upon glucose detection, Rgt2p and Snf3p mediate phosphorylation via casein kinase I (Yck1p/Yck2p) of Mth1p and Std1p, leading to their proteasomal degradation, therefore preventing binding of Rgt1p to the *HXT* promoter regions^11^. Yeast cells can sense a wide range of glucose concentrations and subsequently express transporters required for the amount of glucose present; e.g. low affinity glucose transporters (Hxt1p) when extracellular glucose is high, high affinity glucose transporters (Hxt2p and Hxt4p) when extracellular glucose is low and intermediate glucose transporters (Hxt3p) when extracellular glucose concentrations are neither high nor low^11^. In addition, extracellular glucose concentrations are also detected by the GPCR (G-protein coupled receptor) Gpr1p^12^. In *S. cerevisiae*, the cellular response upon glucose detection is mediated by the cAMP/PKA (protein kinase A) pathway (for an extensive review see ref. 13). The adenylate cyclase Cyr1p is activated by the heterotrimeric Gα protein Gpa2p, which is released from the Gpr1p upon glucose sensing. Cyr1p then increases intracellular cAMP concentrations^14^. Rgt1p is a target of PKA phosphorylation, which causes de-repression of the *HXT* genes. An alternative pathway for the induction of adenylate cyclase activity is via the small GTPases Ras1p/Ras2p, which are activated by phosphorylated glucose^15^. In filamentous fungi, the cAMP/PKA pathway has also been shown to mediate the response to glucose sensing^13^,^16^,^17^. In the presence of glucose, PKA is important for spore germination, hyphal growth, cell wall homeostasis and secondary metabolite production whilst inhibiting alternative carbon source usage^17^,^18^,^19^,^20^.

In contrast to *S. cerevisiae*, glucose sensing and the pathways leading to the transcriptional up-regulation of glucose transporter-encoding genes have not been elucidated in *Aspergillus spp*. and *T. reesei*. To date, several proteins have been described as having a sensory function for non-glucose sugars such as cellobiose and xylose^21^,^22^,^23^. Orthologues of the *S. cerevisiae* glucose sensors Rgt2p and Snf3p have not been described in these fungi. In *A. nidulans*, the G-alpha subunit GanB and the G-protein coupled receptor (GPCR) GprH have been shown to play a role in glucose sensing^24^,^25^. GanB is involved in mediating activation of cAMP synthesis and subsequent PKA activation in the presence of glucose during early conidial germination events^24^. In *Neurospora crassa, rco-3* was shown to be involved in glucose sensing^26^.

The genomes of saprotrophic filamentous fungi encode a large number of transporters responsible for the uptake of different sugars found in plant biomass. Similar to *S. cerevisiae*, a high affinity and a low affinity glucose uptake system have been described for filamentous fungi. In *A. niger*, MstA and MstF were determined to be high affinity glucose transporters whereas *mstC* encodes a low affinity glucose transporter^27^,^28^. In *T. reesei*, STP1 and HXT1 were identified as glucose transporters^29^,^30^. In *A. nidulans*, MstE (AN5860) is a low affinity glucose transporter and HxtA, MstA/HxtD (AN8737) and MstC/HxtB (AN6669) were described as high affinity glucose transporters^31^,^32^,^33^. Additionally, two transporters, HxtC (AN10891) and HxtE (AN1797) have been identified as glucose transporters although it is currently not known with what affinity they transport glucose^32^. In addition to transporting glucose into the cell, these transporters (except for HxtA) are also able to carry other pentose and hexose sugars across the membrane^32^,^33^. Furthermore, HxtA was proposed to be important for sugar metabolism during *A. nidulans* sexual development^31^, whereas deletion of *hxtB* caused a reduction in glucose consumption in the presence of low concentrations of glucose as well as increased resistance to the non-metabolisable glucose analogue 2-deoxyglucose (2DG)^32^. The aforementioned studies^32^,^33^ therefore suggest that HxtB, HxtC, HxtD and HxtE are capable of transporting sugars other than glucose, into the cell whereas HxtA and HxtB may also be involved in fungal developmental processes and/or glucose metabolism as suggested by increased expression of HxtA::GFP during cleistothecia formation^32^ and increased resistance of strain Δ*hxtB* to 2DG^33^.

The aim of this work was therefore to study the transcriptional response of the filamentous fungus *A. nidulans* to high and low concentrations of glucose with the purpose of identifying novel factors involved in glucose sensing and metabolism. This study employed bioreactor-controlled chemostat cultivation, allowing for controlled culture conditions and reduced fluctuation in growth rate and extracellular glucose concentrations^34^. RNA-sequencing was performed on *A. nidulans* grown in low- and high-glucose chemostat conditions. The low-affinity glucose transporter MstC/HxtB was highly induced under glucose-limiting conditions and was shown to be involved in fungal developmental processes, cAMP production and PKA activity. Furthermore, the protein kinase PskA was found to be crucial for glucose uptake and metabolism under high and low glucose conditions.

## Results

### Transcriptional response of *A. nidulans* grown in glucose-limiting and glucose-abundant conditions

**Figure 1:**
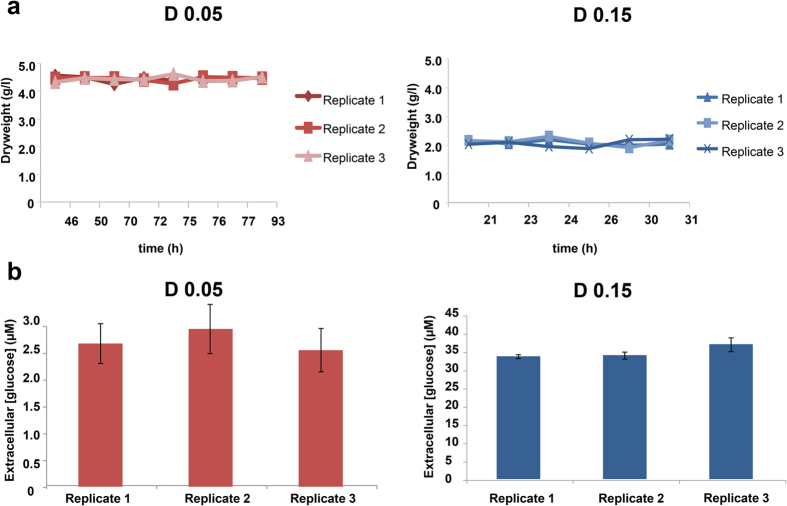
Steady-state growth of *A. nidulans* under low (D = 0.05) and high (0.15) glucose concentrations in chemostat cultures. (**a**) Fungal dryweight and (**b**) residual extracellular glucose concentrations were measured to confirm steady state, which was achieved after ~42 h under low glucose levels and after ~21 h in high concentrations of glucose. All experiments were performed in triplicate. Standard deviations present technical triplicates for each biological replicate.

In order to investigate the transcriptional response of *A. nidulans* in low- and high-glucose conditions, RNAseq of the wild-type strain under continuous batch cultivation was carried out. The dilution rates (D) were set at 0.05 h^−1^ and 0.15 h^−1^ respectively, which corresponded to 25% and 75% of the maximum specific growth rate (μmax, see Materials and Methods). For the lower dilution rate (D = 0.05), the glucose-containing medium was slowly changed (5 l medium/20 h), allowing fungal biomass accumulation and establishing glucose-limiting conditions (~2 μM extracellular glucose), whereas at the higher dilution rate (D = 0.15), the medium was quickly changed (5 l medium/6.5 h) preventing biomass accumulation and allowing high glucose concentrations in the medium (~33 μM extracellular glucose) (Fig. 1a and b). The wild-type strain reached the steady state phase of growth after ~42 h and ~21 h at the lower and higher dilution rates respectively, as determined by measuring the dry weight of samples drawn at different time points from biological triplicates (Fig. 1a). RNAseq was performed on biological triplicates of the wild-type strain in the steady-state phase of growth in low- and high-glucose conditions (from 90–100 h for low and from 30–40 h for high-glucose).

**Figure 2:**
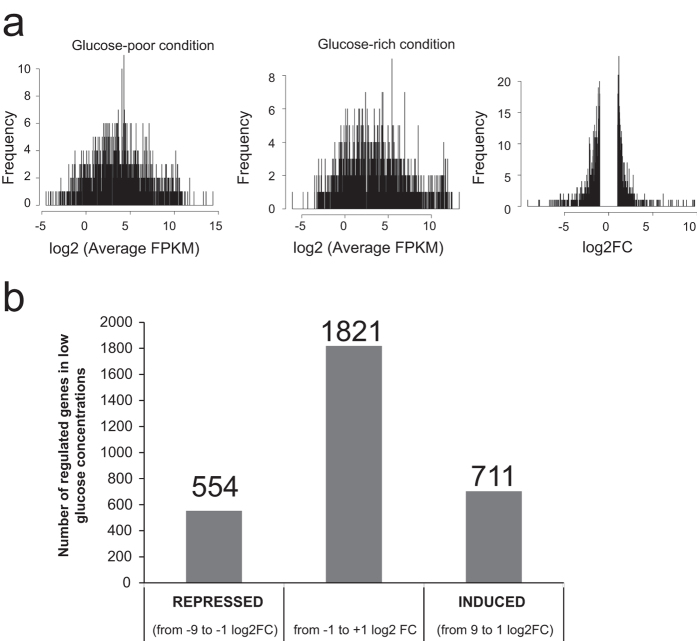
RNA-sequencing of *A. nidulans* when grown in chemostat cultures in the presence of low and high concentrations of glucose. (**a**) FPKM (fragments per kilobase of exon per million mappes reads) and subsequently calculated Log2-fold change (FC) of all the genes under low and high glucose conditions. (**b**) Number of genes which were significantly differently regulated (−1 > log2-FC > 1) in glucose-limiting conditions.

A total of 3086 genes were identified as responding to differences in glucose availability by RNA-sequencing [Supplementary Table S1; False Discovery Rate (FDR) of <0.05]. In this set, 1265 genes had a strong effect, with a fold change of at least 2 times in either direction [log2 fold change <−1 and >1]. Of these, 554 genes were down-regulated (log2 fold change ≤−1) and 711 genes were up-regulated (log2 fold change ≥1), taking as reference the low glucose condition (Fig. 2a and b). The RNAseq data was validated by performing qRT-PCR on 5 randomly chosen transcription factor-encoding genes (Supplementary Figure S1). All genes showed a similar expression pattern as was observed for the RNAseq data.

Gene ontology (GO) enrichment analysis (FetGOat) was performed on the set of 1265 significantly differently regulated genes with log2FC ≤−1 or ≥1. Enrichment of terms involved in catabolic processes of various energy sources such as carboxylic and organic acids, amines, polysaccharides and amino acids was observed under low-glucose conditions. The term “sugar transmembrane transporter activity” was also significantly over-represented (Table 1). A more in-depth analysis of the significantly differently regulated genes showed that genes encoding proteins involved in the glyoxylate cycle (succinate semi-aldehyde dehydrogenase), genes involved in fatty acid synthesis and degradation, ammonium and amino acid uptake, glutamate and glutamine metabolism, aromatic amino acid metabolism, protease production and in the utilization of various intracellular storage compounds (trehalose, starch) as well as acetate metabolism and gluconeogenesis displayed increased expression under low-glucose conditions (Supplementary Table S1).

On the other hand, a transcriptional down-regulation of genes encoding proteins involved in monosaccharide catabolic processes, pyruvate and acetyl-coA metabolic and biosynthetic processes, gene expression, RNA processing and translation was observed in low-glucose conditions (Table 1). A more in depth analysis of these categories showed repression of the gene cluster required for the biosynthesis of the secondary metabolite asperidine A, for quinic acid utilization, fatty acid degradation, genes encoding kinases with roles in pyruvate metabolism, glycolysis and gluconeogenesis as well as amino acid and TCA cycle metabolism (Supplementary Table S1). Altogether, this data suggests that *A. nidulans* responds to low amounts of available glucose with the transcriptional down-regulation of glycolytic and protein biosynthetic genes paralleled by increased transcription of genes predicted to function in the degradation of alternative carbon sources and biosynthesis of secondary metabolites.

Interestingly, 40 genes encoding proteins required for the production of secondary metabolites showed altered expression (26 were up-regulated and 14 were down-regulated) (Supplementary Table S1). Five genes of the monodictyphenone (mdp) biosynthetic gene cluster were induced under low-glucose conditions. In addition, genes involved in the biosynthesis of asparthecin, aspyridone, derivative of benzaldehyde (dba), and sterigmatocystin, were also up-regulated (Supplementary Table S1). Asparthecin, aspyridone (cytotoxic), sterigmatocystin (carcinogenic) and mdp are polyketides synthesized from acyl-CoA units and are thought to provide *A. nidulans* with a competitive advantage in the soil; some have gained medical interest due to their cancer preventive and anti-bacterial properties^35^. Furthermore, the gene encoding a polyketide synthase involved in the formation of the conidial green pigment was also significantly up-regulated under glucose-limiting conditions (Supplementary Table S1).

To further analyse the transcriptional response of *A. nidulans* to glucose, the RNAseq dataset comprising genes exclusively expressed (or exclusively induced) in either low-glucose or high-glucose conditions, e.g. which were condition-specific, were analysed. Considering the FPKM value higher than 1 in low or high glucose conditions (p < 0.00005), a total of 4 genes were specifically modulated in high glucose concentrations and 10 genes were specifically up-regulated in low glucose conditions (Supplementary Table S1). Interesting, genes encoding transporters, monooxygenases, proteins involved in secondary metabolism, methyltransferases as well as uncharacterized proteins were specifically up regulated in low-glucose conditions. Additionally, in high-glucose conditions, genes encoding a protein with a role in conidium formation, another encoding a putative alpha-1,3- glucanase (agnE), and two genes not yet characterized were also up-regulated.

### The hexose transporter-encoding gene *hxtB* is highly up-regulated under glucose-limiting conditions

**Figure 3:**
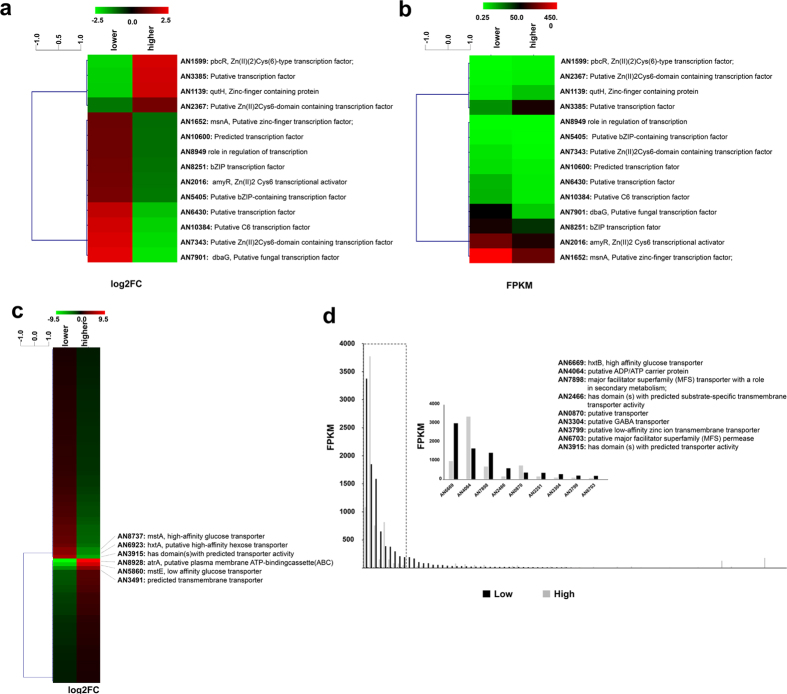
Heat maps of significantly differently regulated transcription factor and transporters. (**a**,**b**) transcription factor, (**c**,**d**) transporter encoding genes according to the Log2 fold-change (**a,c**) or FPKM (fragments per kilobase of exon per million mappes reads) (**b**,**d**) values.

With our particular interest in glucose signaling and transport, the RNAseq data was mined for differential expression of genes encoding known or predicted transcription factors and transporters between low- and high-glucose conditions. A total of 14 putative transcription factor-encoding genes were differentially expressed under both conditions (Fig. 3a and b). Of these, 10 displayed increased and 4 displayed decreased expression under low-glucose conditions (Fig. 3a, Supplementary Table S1). Among the 10 up-regulated genes, three encode the previously, partially, characterized transcription factors MsnA (AN1652), AmyR (AN2016) and DbaG (AN7901) whereas the remaining six encode putative, uncharacterized transcription factors. MsnA was shown to be induced under high salt conditions and is thought to be targeted by the HOG (high osmolarity glycerol) pathway^36^. AmyR is involved in the induction of amylolytic genes required for starch utilization^37^, whereas DbaG is a transcription factor that belongs to the dba secondary metabolite gene cluster (see previous results section). Of the four down-regulated genes in low-glucose conditions, two encode the transcription factors QutH (AN1139) and PbcR (AN1599) whereas the remaining two encode putative, uncharacterized transcription factors (Fig. 3a). QutH is a zinc finger-containing protein of the quinic acid utilization gene cluster, whereas PbcR is a transcription factor which activates the diterpene compound gene cluster responsible for the production of the secondary metabolite ent-pimara-8(14), 15-diene. Like the polyketides (see above), the terpenes are another group of secondary metabolites that are of particular interest due to their pharmaceutical properties^38^. The modulation of the same transcription factor-encoding genes is also shown in absolute FPKM (fragments per kilobase of exon per million mapped reads values) (Fig. 3b) and results are the same as presented in Fig. 3a.

A total of 89 transporter-encoding genes were significantly modulated in low-glucose conditions (Fig. 3c and d). Of these, 33 genes were down-regulated and 56 were up-regulated. Genes encoding the two transporters AtrA and MstE, presented the highest down-regulation (log2 fold-change of −9.47 and −7.89 respectively). The former is involved in multidrug resistance^39^, whereas the latter has previously been characterized as a low affinity glucose transporter^40^. The transporter-encoding genes *hxtA, hxtD/mstA* and AN3915 (not yet characterized) presented the highest up-regulation among the 56 genes (Fig. 3c). HxtA and HxtD/MstA have previously been described as high affinity glucose transporters^31^,^32^,^33^. The expression of *hxtB* was the highest among all transporter-encoding genes in glucose-limiting conditions, when looking at the data in absolute FPKM values (Fig. 3d). Although *hxtB* was previously shown to be highly up-regulated in low glucose (0.1%) concentrations during batch cultivation^32^, this work shows that it is also greatly induced during chemostat cultivation. In summary, the analysis presented here identified several putative, uncharacterised transcription factor and transporter-encoding genes.

### HxtB is a low affinity glucose transporter and localizes to the plasma membrane in glucose starvation conditions

**Figure 4:**
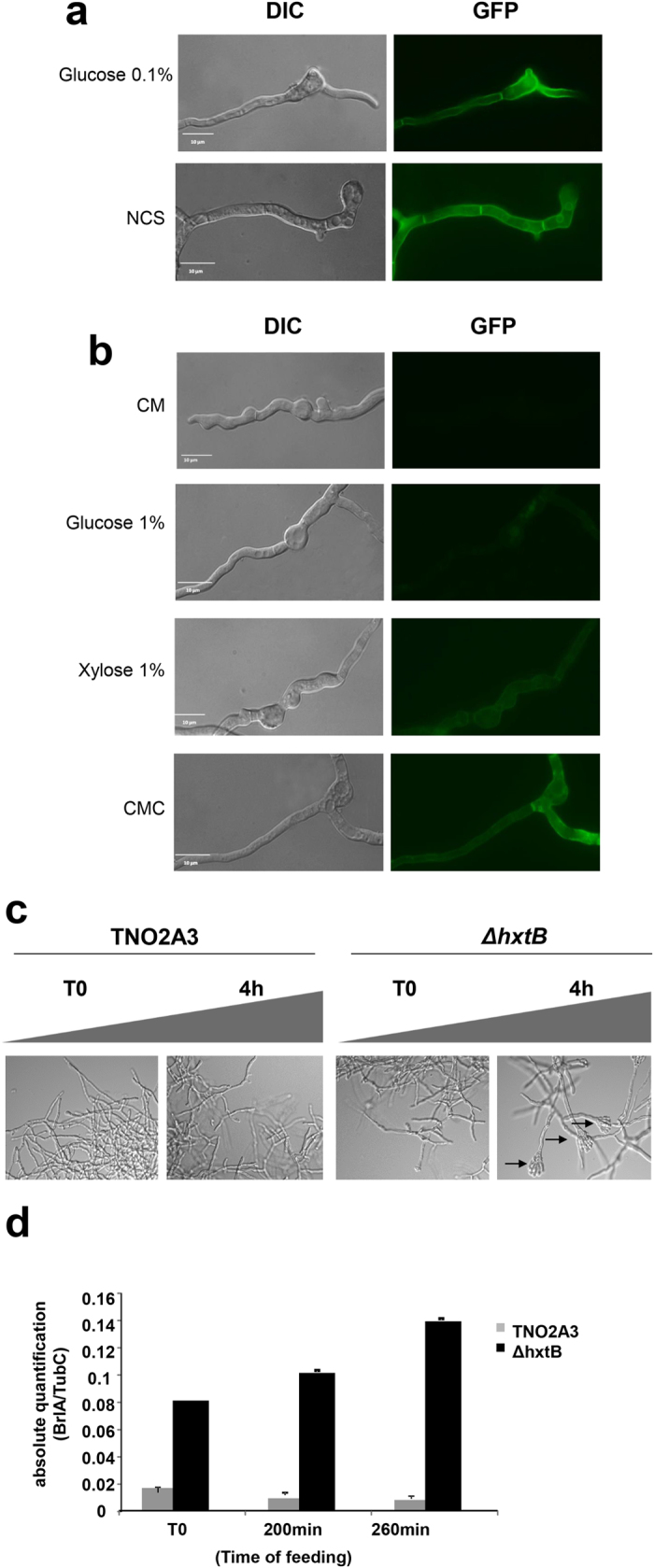
HxtB is involved in fungal developmental processes. (**a**,**b**) Microscopy of the HxtB::GFP strain in the presence of different glucose concentrations and carbon sources. (**c**) Microscopy of the wild-type and Δ*hxtB* strains after 4 h chemostat cultivation. Arrows indicate conidiophore structures. (**d**) Expression of the developmental regulator *brlA*, as determined by qRT-PCR, in the wild-type and Δ*hxtB* strains after 4 h chemostat cultivation.

The RNAseq data presented here identified the transporter HxtB as up-regulated under low-glucose conditions, with the largest expression among all transporter-encoding genes. HxtB was previously identified as a xylose and glucose transporter with a possible role in glucose metabolism^31^,^38^. Deletion of *hxtB* in *A. nidulans* caused a reduced affinity for glucose and was therefore classified as a high affinity glucose transporter^32^,^33^. To further characterize the glucose affinity of HxtB, it was heterologously expressed in *S. cerevisiae* strain EBY.VW4000 as described previously^32^. Glucose uptake of the HxtB-expressing *S. cerevisiae* strain was measured and Km and Vmax values were derived (Supplementary Figure S2). HxtB was found to be a low affinity glucose transporter with a Km of ~15 mM which is similar to the Km (~10 mM) of the *S. cerevisiae* intermediate glucose transporter Hxt2^41^. To confirm HxtB expression, the protein was tagged with GFP. The *hxtB* gene was replaced with the *hxtB*::*gfp* fragment under the control of the native promoter and strain construction was confirmed by PCR before cellular localization of HxtB::GFP was microscopically assessed. In the presence of low concentrations of glucose (0.1% w/v) and when inoculated in media without any carbon source (starvation), a strong fluorescent signal of HxtB::GFP was observed in the hyphal membranes and septa (Fig. 4a). On the other hand, when the HxtB::GFP strain was inoculated in complete medium (2% w/v glucose), 1% w/v glucose and in the presence of 1% w/v of the pentose sugar xylose, fluorescence was present but weak (Fig. 4b), indicating a reduced accumulation of HxtB::GFP in the fungal membranes in these conditions. A somewhat stronger fluorescence was also observed when hyphae were inoculated in the presence of the complex carbon source carboxymethylcellulose (CMC) (Fig. 4b). These results indicate HxtB is a low affinity glucose transporter which is strongly expressed under glucose-limiting conditions in agreement with previous studies^32^,^33^.

### Deletion of HxtB causes a hyperconidiation phenotype under continuous supply of low and high amounts of glucose

In order to further evaluate its glucose-transporting capabilities and any involvement in glucose metabolism, the Δ*hxtB* strain^32^ was grown in low- and high-glucose conditions in chemostat cultures as described above for the wild-type strain. In contrast to the wild-type strain, the *hxtB* deletion mutant started to conidiate heavily, turning the bioreactor dark green after 24 h of continuous cultivation under low- (D0.05, ~2 μM glucose) and high glucose (D0.15, ~33 μM) concentrations conditions (data not shown). To confirm the hyperconidiating phenotype, samples were taken from both the wild-type and Δ*hxtB* chemostat cultures after 4 h at the high dilution rate (*i.e.*, high-glucose conditions) and analysed by microscopy (Fig. 4c). In addition, qRT-PCR of *brlA*, encoding a transcription factor involved in conidiophore development^14^, was performed under the same conditions (Fig. 4d). Indeed, the Δ*hxtB* strain formed conidiophores after 4 h chemostat cultivation and the expression of *brlA* was almost 10 times higher in this strain than when compared to the wild-type strain (Fig. 4c and d).

Unfortunately, this hyperconidiating phenotype prevented reliable RNAseq analysis on the Δ*hxtB* strain when cultivated under chemostat conditions. Interestingly, we have not observed this conidiation phenotype during batch cultivation, where the Δ*hxtB* strain was grown for 32 h in 0.1% w/v glucose or for 72 h in 1% w/v glucose^32^. Taken together, these results indicate that HxtB potentially regulates the induction of asexual development in high and low-glucose conditions under chemostat cultivation in *A. nidulans*.

### HxtB is involved in cAMP accumulation, protein kinase A activity and the Ras signaling pathway

**Figure 5:**
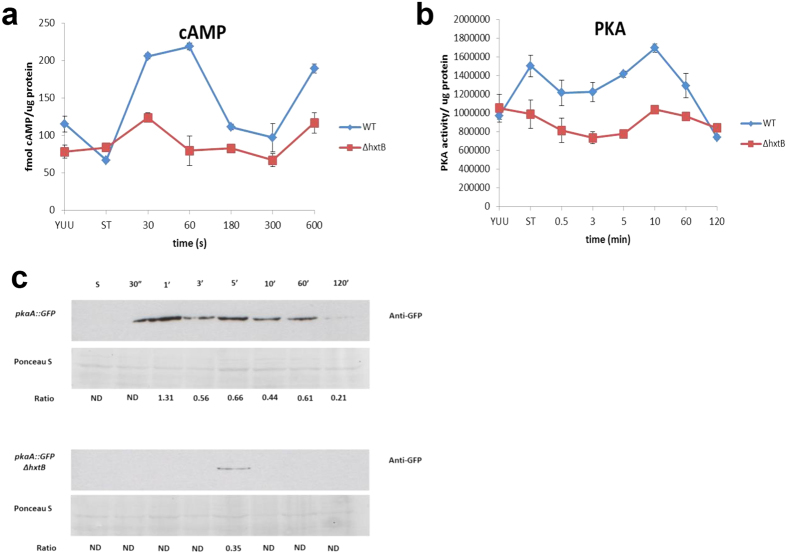
HxtB is involved in glucose signalling events. cAMP levels (**a**) and PKA (**b**) activities were measured in the wild-type and Δ*hxtB* strains when grown for 24 h in complete medium and after the addition of glucose for the indicated amounts of time. (**c**) Western blot of PkaA::GFP in the wild-type and Δ*hxtB* strains when grown in the same conditions as described above.

The results described above suggest that HxtB may be involved in cellular signalling events that control fungal developmental processes. C^14^-glucose transport experiments revealed a significant delay in glucose transport in the *hxtB* null mutant under high- and low-glucose concentrations, although eventually this strain consumed all extracellular glucose^32^. The increased conidiation phenotype of this mutant in high and low-glucose conditions suggests that HxtB could play a role in glucose signalling events. The cellular response to glucose sensing is mediated by the cAMP/PKA pathway^13^,^15^. Therefore, cAMP accumulation and PKA activity were determined in both the wild-type and the Δ*hxtB* strains. Both strains were grown overnight in complete medium before being transferred to minimal medium without any carbon source for 4 h. Glucose was then added to a final concentration of 2% w/v for different amounts of time before cAMP concentrations and PKA activities were measured (Fig. 5a and b). In the wild-type strain, addition of glucose caused an immediate increase of cAMP which thereafter oscillated between high and low amounts and which was absent in the Δ*hxtB* strain (Fig. 5a). Similarly, addition of glucose increased PKA activity in the wild-type strain but not in the Δ*hxtB* strain (Fig. 5b). In agreement, Western blot analysis of the PkaA::GFP (Supplementary Table S2) and the PkaA::GFP Δ*hxtB* (Supplementary Table S2) strains when grown in the same conditions as described for Fig. 5a and b, showed accumulation of PkaA after the addition of glucose in the wild-type strain but not in the Δ*hxtB* strain (Fig. 5c). These results indicate that HxtB influences glucose-related, downstream signalling events, although we cannot completely discard the possibility that a reduction of intracellular glucose concentrations due to a delay in glucose transport may also contribute to the observed phenotype.

An alternative pathway for increasing cellular cAMP concentrations is via Ras signalling^15^. RasA is a small GTPase involved in the control of fungal developmental processes such as spore swelling and germination, formation of vegetative and aerial hyphae and conidiation^42^. The RasA^G17V^ strain contains a mutation in the *rasA* gene (glycine to valine, position 17), which causes constitutive activation of RasA by locking it in its GTP-bound form^42^. Because *rasA*^G17V^ is under the control of the *alcA* promoter, cyclobutanone was used as a non-metabolisable, inducing compound^43^. In order to gather additional evidence for the involvement of HxtB in fungal developmental processes, the wild-type, RasA^G17V^, Δ*hxtB* and RasA^G17V^ Δ*hxtB* strains were inoculated for 16 h in minimal medium without any carbon source and microscopically examined before the percentage of germinated spores was calculated for each strain. As expected, conidia of the wild-type strain had a very low germination rate (~15% germination) in no carbon source conditions. On the other hand, conidia of the RasA^G17V^ and Δ*hxtB* strains presented increased germination (~40% and ~55% respectively) when compared to the wild-type strain (Supplementary Figure S3B). The RasA^G17V^Δ*hxtB* strain presented the highest percentage of germination (~70%), which suggests that HxtB and RasA function in parallel interacting pathways to regulate spore germination (Supplementary Figure S3B).

### The protein kinase PskA is important for glucose uptake and metabolism

Protein kinases catalyse the phosphorylation of cellular proteins, therefore regulating the function, structure and/or localisation of their target proteins^44^. They play an important role in the regulation of most cellular processes including signalling pathways. To further understand the response of *A. nidulans* to the presence of high- or low-glucose concentrations, the RNA dataset was screened (including not significantly differently regulated genes) for protein kinase-encoding genes. The PAS (Per-Arnt-Sim) domain-containing serine/threonine protein kinase PskA (AN4536) had a higher expression in low-glucose conditions (FPKM 13.8 low-glucose vs FPKM 9.0 high-glucose), although the difference was not statistically significant. In *S. cerevisiae*, there are two paralogues, termed *PSK1* and *PSK2* that arose from whole genome duplication. These two kinases are important for sugar metabolism, protein translation and glycogen biosynthesis, and thus play an important role in sugar consumption and storage and protein translation^45^. Likewise, PskA is predicted to be involved in the repression of glycogen synthesis and in (1–6)-beta-D-glucan biosynthesis in *A. nidulans*. Based on the aforementioned studies, we decided to characterize the effect of *pskA* deletion on growth, glucose uptake and intracellular glycogen and trehalose concentrations in *A. nidulans*.

**Figure 6:**
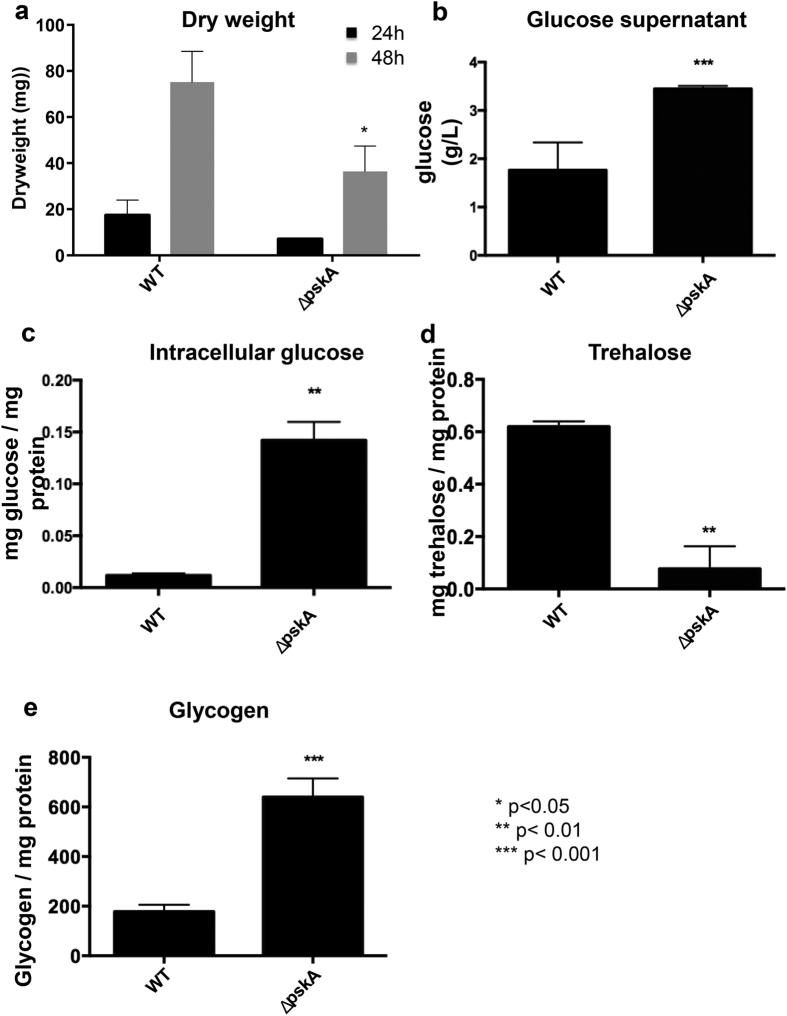
PskA is important for intracellular and extracellular glucose utilisation. Fungal dry weight (**a**), extracellular (**b**) and intracellular (**c**) glucose concentrations as well as intracellular trehalose (**d**) and glycogen levels (**e**) were measured in the wild-type and Δ*pskA* strains when grown for 24 h in complete medium and then transferred to minimal medium supplemented with glucose for 16 h.

Growth of the wild-type and the Δ*pskA* strains (Supplementary Table S2) were characterized when grown for 24 h and 48 h in minimal medium supplemented with 1% w/v glucose. Biomass accumulation was severely reduced in the Δ*pskA* strain when compared to the wild-type strain (Fig. 6a). Glucose uptake, as determined by measuring the residual glucose in the culture supernatants, was reduced in the Δ*pskA* mutant, suggesting difficulties with glucose transport (Fig. 6b). Furthermore, accumulation of intracellular glucose and glycogen (Fig. 6c and e) coupled with reduction in intracellular trehalose levels (Fig. 6d) in the Δ*pskA* strain imply that a general deregulation of glucose metabolism has occurred in the Δ*pskA* mutant. These results suggest an important role for PskA in glucose metabolism, including uptake and the generation of intracellular storage compounds.

## Discussion

One of the drawbacks for the process of 2G biofuel production from plant lignocellulosic biomass is the inability of filamentous fungi to secrete key enzymes required for lignocellulose hydrolysis in the presence of glucose. Filamentous fungi such as *T. reesei* and *Aspergillus spp*., which are currently being investigated for hydrolytic enzyme production, prefer consuming simple, readily metabolized sugars such as glucose rather than alternative, more complex carbon sources such as those contained within lignocellulose; this preference is known as carbon catabolite repression (CCR) and is mediated by the transcription factor CRE1/CreA^6^. Although CRE1/CreA-mediated repression of genes encoding enzymes for lignocellulose degradation has been extensively studied^6^,^7^,^8^,^9^,^10^, far less is known about how glucose is sensed and the corresponding downstream signalling events. The aim of this work was therefore to analyse the response of *A. nidulans* to low- and high-glucose conditions with the purpose to identify novel factors involved in glucose sensing and metabolism.

The genome-wide transcriptional response of *A. nidulans* when grown in the presence of low and high concentrations of glucose was assessed by RNA-sequencing (RNAseq). Continuous (chemostat) cultivation was performed which allowed for controlled culture conditions with reduced fluctuation in growth rate and extracellular glucose concentrations^33^,^46^. Furthermore, *A. nidulans* pellet formation was prevented allowing for homogenous, macroscopic morphology and glucose uptake throughout the culture. Continuous chemostat cultivation has been carried out for several filamentous fungi such as *A. niger*^34^, *T. reesei*^47^, *Penicillium chrysogenum*^48^ and *N. crassa*^49^ but until now has not been published for *A. nidulans*. This study shows that continuous batch and chemostat cultivation can also be applied to *A. nidulans*, which is considered an important ‘reference’ organism for studying various cellular processes in filamentous fungi.

In low-glucose conditions, there was an over-representation of Gene Ontology terms involved in catabolic processes of different, non-monosaccharide energy sources whereas pyruvate and acetyl-coA-related processes as well as protein synthesis were under-represented. These results indicate that the fungus is adapting to severe glucose limitation. In high-glucose conditions, genes encoding proteins involved in the same biological processes were modulated. Several transcription factor- and transporter-encoding genes that have not been characterized were identified as being significantly up-or down-regulated in low- and high-glucose conditions. These genes are potential targets for future detailed investigation of the transcription factors involved in cellular responses to starvation and/or glucose-abundant conditions as well as identifying additional sugar transporters that could be useful for biotechnological applications.

Furthermore, there was also a substantial regulation of genes encoding transcription factors and enzymes required for the biosynthesis of various secondary metabolites (SM). In particular, genes involved in polyketide synthesis, such as asparthecin, aspyridone, derivative of benzaldehyde (dba) and sterigmatocystin were induced whereas genes encoding proteins involved in aspernidine A, emericellamide, terpene biosynthesis and quinic acid utilization were down-regulated. An up-regulation of secondary metabolite production under carbon starvation conditions has been previously described in *A. nidulans*^50^ and *A. niger*^51^. Nutrient limitation is accompanied by fungal developmental changes such as asexual structure formation and sporulation^25^,^51^. Polyketide synthases are essential for the production of secondary metabolites but have also been shown to be important for melanin production, a pigment pre-dominantly found in the conidia of *Aspergillus spp* and which protects against environmental stresses such as UV light and radiation^52^,^53^,^54^. Indeed, several polyketide synthase-encoding genes of different secondary metabolite clusters were up-regulated in low-glucose conditions in *A. nidulans*, including one polyketide synthase predicted to be involved in the formation of the conidial green pigment. The up-regulation of polyketide-type secondary metabolites under chemostat conditions could be due to the development of asexual structures. However, as we have not observed the formation of asexual structures during chemostat cultivation of wildtype *A. nidulans*, it is likely that the function of the produced secondary metabolites is linked to the survival of the fungus in a competitive, nutrient-limited environment as has been proposed previously^35^.

One of the transporter-encoding genes which was up-regulated under chemostat carbon starvation conditions and whose expression level (FPKM) was highest among all transporter-encoding genes, was HxtB. HxtB was previously described as a high affinity glucose transporter^32^,^33^ although heterologous expression in *S. cerevisiae* determined it to be a low affinity transporter. This discrepancy is likely due to the different systems used to characterize this transporter (e.g. deletion in a filamentous fungus vs. heterologous expression in a yeast). The expression of *hxtB* also increased during batch cultivation at low-glucose concentrations^32^, suggesting that HxtB is important for growth in glucose-limiting concentrations during different cultivation conditions. HxtB was previously confirmed to be a glucose transporter that also accepts other monosaccharides, and deletion of *hxtB* resulted in delayed glucose uptake as well as increased resistance to the non-metabolizable glucose analogue 2-deoxy glucose (2DG)^32^. These results indicate that HxtB may be involved in cellular processes other than sugar transport. In agreement, this study found that deletion of *hxtB* caused hyperconidiation in the chemostat cultures under high and low glucose concentrations, a phenotype that was not observed during batch cultivation. One important difference between both cultivation modes is that batch cultivation (irrespectively whether this is performed in shake flasks, bioreactors or during cultivation on agar plates) results in complete consumption of the initially added glucose and therefore adjusts carbon starvation after prolonged cultivation, whereas chemostat cultivations only limit the available glucose to a certain amount but provide the fungus with a continuous supply. It is therefore likely that the low amounts of glucose provided here during chemostat cultivation provide the necessary energy for increasing conidiation of the Δ*hxtB* strain. The deletion of *hxtB* was paralleled by an increased mRNA accumulation of the transcription factor *brlA*, which has been shown to play a central role in conidiophore development^14^,^55^. This is in contrast to a previous study where the expression of *hxtB* was found to decrease during sexual and asexual development^32^. This discrepancy may be due to the differences in the experimental setup (chemostat cultivation vs. solid media incubation). In summary, these results suggest a role for HxtB in asexual developmental processes during continuous chemostat cultivation in high- and low-glucose conditions.

In addition, the deletion of *hxtB* caused reduced cAMP levels and PKA activity suggesting a role for this transporter in downstream glucose signalling events, although it cannot be excluded that this may be due to delayed glucose uptake under high- and low-glucose conditions, as was previously shown for this strain^32^. The Δ*hxtB* mutant is able to transport glucose^32^ and it is currently not known whether a certain threshold of intracellular glucose concentration triggers a spike in cAMP levels and PKA activity. Therefore, further studies are required to confirm/reject a role of HxtB in glucose sensing and signaling. In *T. reesei* and *N. crassa*, several transporters have been identified to play a role in cellulose sensing and to be important for regulating downstream signaling events that lead to proper utilization of this carbon source^21^,^22^. Studies on glucose sensing and subsequent activation of downstream targets are very limited in *Aspergillus spp*. but it is possible that, like in *T. reesei* and *N. crassa*, carbon source transporters can exert a sensory and regulatory function for the utilization of glucose. Furthermore, HxtB was also shown to be involved in RasA-mediated glucose signaling. Still, the molecular mechanism by which HxtB regulates cAMP levels and RasA activity remains subject to future studies.

This study also identified the protein kinase encoded by AN4536 as being important for glucose metabolism. We chose to name this protein kinase PskA due to its homologue Psk1p of *S. cerevisiae*. A pseudo-kinase has also been previously named PskA^56^. Although the two homologues of PskA have been characterized in *S. cerevisiae*^45^, direct evidence for a role of PskA in glucose metabolism in *A. nidulans* is absent. This study found that *pskA* was expressed in both high- and low-glucose chemostat conditions. Furthermore, *pskA* was shown to be important for glucose uptake as well as the correct accumulation and utilization of intracellular storage compounds such as glycogen and trehalose. These results indicate that, like in *S. cerevisiae*, PskA may have a similar function in the control of sugar flux and metabolism in *A. nidulans*. PskA is therefore an interesting target for future carbon metabolism-related studies focused on elucidating cellular signalling pathways that could potentially be manipulated for biotechnological applications.

In conclusion, this study presents a detailed analysis of the genome-wide transcriptional response of *A. nidulans* to low- and high-glucose conditions using continuous chemostat cultivation. Several transcription factor- and sugar transporter-encoding genes were identified as being important for this response, representing novel, potential targets for future studies. The glucose and xylose transporter HxtB was shown to be important for downstream glucose signalling events and fungal developmental processes that suggest a transceptor-like role for this protein. Furthermore, the protein kinase PskA was also identified as being important for glucose-metabolism related processes. This study therefore lays a basis for further investigation of how biotechnologically important filamentous fungi respond to glucose, a carbon source which represses the production of industrially relevant enzymes from these organisms.

## Methods

### Strains and growth conditions

All strains (Supplementary Table S2) were grown at 37 °C (except where stated) in minimal medium or complete medium with (solid) or without (liquid) 2% w/v agar as previously described^32^. When necessary, uridine/uracil (1.2 g/L each) and/or pyridoxine (0.5 μg/mL) were added. For the batch and chemostat cultures, *A. nidulans* was grown for 3 days at 30 °C on plates containing minimal medium supplemented with glucose. Conidia were harvested with a 0.9% (w/v) NaCl solution and washed twice in the same solution. Batch and chemostat cultivations were performed using Ammonium Minimal Medium (4.5 g/L NH_4_Cl, 1.5 g/L KH_2_PO_4_, 0.5 g/L KCl, 0.5 g/L MgSO_4_ × 7H_2_O, 1 ml trace metal solution, 160 mg uridine, pH 3.0).

All fungal biomass was separated from the culture medium by vacuum filtration and snap-frozen in liquid N_2_. The biomass was subsequently used for RNA/DNA extractions, biochemical assays or freeze-dried and weighed to determine the dry weight.

### Batch and chemostat cultures

Chemostat cultures were performed in the BioFlo310 Fermentor/Bioreactor (6.6 l, New Brunswick Scientific, NJ, USA) as described previously with modifications^51^. First, batch cultivation of the *A. nidulans* FGSC A4 strain was carried out in 5 kg ammonium-supplemented minimal media which contained 1% w/v glucose and 0.003% w/v yeast extract. A total of 10^9^ spores/L was inoculated and left to germinate for 8 h at 30 °C, 250 rpm whilst keeping the pH at 3.0. Polypropyleneglycol (PPG) was then added as an antifoam agent. The agitation was increased to 600 rpm before 20 g of NaOH were added, increasing the pH gradually from 3.0 to 5.5 over a time period of 4 h. The chemostat cultivation, using the same growth medium, was then started in the late-exponential growth phase of the fungus. The dilution rates (D) were set at 0.05 h^−1^ (lower) and 0.15 h^−1^ (higher). NaOH addition (to prevent acidification of the medium by the fungus) and CO_2_ production data were used to define the maximum specific growth rate (μmax) which was 0.20 h^−1^ for the wild-type strain (data not shown). Steady state of growth was reached after ~45 h for the lower dilution rate and ~20 h for the higher dilution rate. Steady state [specific growth rate (μ) is equal to the dilution rate] was determined by monitoring CO_2_ production, O_2_ consumption, glucose consumption and by the addition of NaOH. All experiments were carried out in triplicate.

### Determination of extracellular and intracellular glucose concentrations

Residual glucose in the supernatants of the chemostat cultures was determined enzymatically using the hexokinase and glucokinase method as previously described^57^.

Intracellular and extracellular glucose concentrations in non-chemostat cultures were measured using the Glucose GOD-PAP Liquid Stable Mono-reagent kit (LaborLab Laboratories Ltd. Guarulhos, São Paulo, Brazil), according to the manufacturer’s instructions. For the determination of intracellular glucose concentrations, whole cell extracts were generated by re-suspending mycelial powder in 1 mL of extraction buffer [50 mM Tris base pH 7.0, 50 mM NaF, 1 mM NaVO_3_, 1 mM DTT, phosphatase inhibitor cocktail P0044 (Sigma) and an EDTA-free protease inhibitor cocktail (Roche)], followed by centrifugation and removal of the glucose-containing supernatants.

### RNA extraction and qRT-PCR

Total cellular RNA was extracted using TRIZOL (Invitrogen) according to manufacturer’s instructions. DNA was degraded with DNAse (Promega) according to manufacturer’s instructions and RNA was subsequently purified using the RNeasy^®^ Mini Kit (Qiagen), according to manufacturer’s instructions. The quality of the RNA was verified using the Agilent Bioanalyser 2100 (Agilent technologies) and a RIM value of 8.0 as the RNA quality threshold. cDNA was synthesized from RNA using the ImProm-II™ Reverse Transcription System (Promega) according to manufacturer’s instructions. qRT-PCR was performed using the ABI 7500 Fast Real-Time PCR System (Applied Biosystems, Foster City, CA, USA) and the SYBR Green PCR Master Mix kit (Applied Biosystems), according to manufacturer’s instructions. Reactions and calculations were performed as previously described^58^ and gene expression were normalised by the expression of *tubC*. Primer sequences used in this study are listed in Supplementary Table S3.

### Library preparation and RNA sequencing

RNA was extracted from 100 mg of ground mycelial powder of the wild-type strain, when grown in biological triplicates in chemostat cultures during the steady state phase of growth, using the Qiagen RNeasy minikit according to the manufacturer’s instructions. RNA quality was checked as described above.

RNA-seq libraries were prepared using Illumina TruSeq RNA library Prep kit v2 kit, which uses polyA-based mRNA enrichment. Sequencing was carried out in a HiSeq2000 using paired-end (2 × 50 bp) chemistry. Each condition was evaluated with three biological replicates. Initial quality check was carried out with FastQC (http://www.bioinformatics.babraham.ac.uk/projects/fastqc/), followed by cleaning step in Trimmomatic v.0.32^59^. Reads were mapped using Bowtie 2^60^ and Tophat2^61^ against the *Aspergillus nidulans* genome assembly version s10-m03-r07 (release on 02-Mar-2014, available in the AspGD). Saturation of known splice junctions was verified using the junction_saturation function from the RseQC package^62^, in order to check for proper sequencing depth. Transcriptome analysis was carried out using Cufflinks v.2.1 as follows: transcript assembly and quantification for each library was performed with Cufflinks^63^, followed by generation of a master transcriptome assembly by merging all libraries with cuffmerge. Transcript differential expression (DE) tests were carried using cuffdiff. Differentially expressed transcripts with q-values < 0.05 (adjusted p-values) were considered significant. The R package CummeRbund (http://compbio.mit.edu/cummeRbund/) was used for DE quality assessment (e.g. identification of outlier replicates). The RNAseq data was submitted as raw fastq files to the NCBI Short Read Archive (SRA) SRP090936, BioSamples SAMN05876028 (low glucose) and SAMN05876029 (high glucose) associated with BioProject PRJNA345604 (http://www.ncbi.nlm.nih.gov/bioproject/345604).

### Trehalose assay

Trehalose concentrations were determined in 10 μg of total extracted cellular protein as described previously^64^. Mycelia were grown for 24 h in 50 ml complete medium or after transfer to minimal media supplemented with 1% glucose for an additional 16 h.

### Glycogen assay

Mycelial glycogen concentrations were measured as previously described by ref. 65 with modifications. Mycelial powder was re-suspended in 1 mL of 80% ethanol and incubated for 1 min at 37 °C. Samples were centrifuged for 5 min at 14.000 g, before the pellets were re-suspended in 1 mL of 0.25 M Na_2_CO_3_. Samples were incubated for 90 min at 100 °C and then cooled down to room temperature. 200 μL of each sample was mixed with 50 μL of 3 M acetic acid and then with 700 μL of 0.2 M acetic acid pH 4.8. The glycogen within the samples was de-branched by incubating it with 10 μL of amyloglucosidase 75U solution (Sigma A7420) at 37 °C for 20 h. Reactions were stopped at 95 °C for 5 min and samples were precipitated by centrifugation at 14.000 g for 5 min at 4 °C. 20 μL were then used to measure the amount of free glucose using the Glucose GOD-PAP Liquid Stable Mono-reagent kit, according to manufacturer’s instructions.

### PKA activity and cAMP concentration

A total of 1 × 10^7^ conidia were inoculated in complete media for 16 h, 37 °C, 180 rpm before being transferred to minimal media containing no carbon source for 4 h in the same conditions. Glucose was then added to a final concentration of 2% (w/v) for the indicated amounts of time. Cellular protein extracts were prepared according to ref. 20. A Bradford Assay was carried out to quantify the protein concentration in the samples (Bio-Rad, according to manufacturer’s instructions). cAMP concentrations and PKA activities were measured in the cell extracts using the Amersham cAMP Biotrak EIA system assay (GE Healthcare) and the Peptag cAMP dependent PKA activity assay (Promega) according to manufacturer’s instructions. The phosphorylated substrate was then run on a 1% w/v agarose gel and the intensity of the band was quantified using ImageJ. All samples were normalized by intracellular protein concentrations.

### Western blotting

Mycelia were grown in the specified conditions and ground to a fine powder under liquid N_2_ before being mixed with extraction buffer [50 mM Tris–HCl pH 7.0, 50 mM NaF, 1 mM NaVO_3_, 1 mM DTT, phosphatase inhibitor cocktail P0044 (Sigma) and the complete mini EDTA-free protease inhibitor cocktail (Roche)]. Samples were centrifuged for 5 min at 14000× *g* and the protein concentration in the supernatant was determined as described above. 50 μg of total protein were run on pre-made gels, before being transferred to a membrane as described previously^10^. Membrane blocking and washes, primary and secondary antibody incubation as well as membrane signal detection were carried out as described previously^10^. The primary rabbit polyclonal IgG antibody anti-GFP (Abcam #ab290) was used in a 1:10000 dilution whereas a 1:5000 dilution was used for the secondary anti-rabbit IgG HRP linked antibody (Cell Signaling Technology, Beverly).

### Microscopy studies

For fluorescent microscopy, 1 × 10^5^ conidia were inoculated in 3 ml minimal medium supplemented with the respective carbon source, in small petri dishes containing a glass cover slip. Conidia were left to germinate overnight at 25 °C before coverslips were washed with 1 × PBS (137 mM NaCl, 2.7 mM KCl, 10 mM Na_2_HPO_4_, 1.8 mM KH_2_PO_4_) and either viewed under the microscope or transferred to minimal medium without any carbon source. All slides were viewed with a Carl Zeiss (Jena, Germany) microscope, equipped with a 100 W HBO mercury lamp epifluorescence module, using the 100x magnification oil immersion objective lens (EC Plan-Neofluar, NA 1.3). Phase contrast brightfield (DIC) and fluorescent images (GFP, DAPI) were taken with an AxioCam camera (Carl Zeiss), and images were processed using the AxioVision software version 3.1 and saved as TIFF files. Further processing was performed using Adobe Photoshop 7.0 (Adobe Systems Incorporated, CA).

To determine the germination capacity of the wild-type, *rasA*^*G17V*^, *ΔhxtB* and *rasA*^*G17V*^ *ΔhxtB* strains, 1 × 10^5^ conidia were inoculated into small petri dishes containing sterile coverslips and 3 ml no carbon source minimal medium supplemented with 10 μM cyclobutanone. Cells were incubated at 37 °C for 14 h before being viewed under the microscope. A minimum of 100 germinated and non-germinated conidia was counted. The experiment was carried out in triplicate.

### *S. cerevisiae* glucose uptake assay

The glucose uptake of the HxtB-expressing *S. cerevisiae* strain^32^ was assessed by the incorporation of D-[U-^14^C]glucose [289.0 mCi/mmol (10.693 GBq)/mmol]; Perkin Elmer Life Sciences] in different D-glucose concentration. Briefly, 500 ml of SC-Trp medium supplemented with glucose was inoculated at 30 °C with the EBY.WV4000 strain harbouring the *hxtB* gene^32^. Culture started from an initial OD_600_ 0.1 and were grown until reach OD_600_ ~ 0.6. Cells were harvested by centrifugation (4000 rpm), washed twice with 50 mL ice-cold water and resuspended in 1250 μL of cold water. A total of 400 μL of cells was then diluted in 800 μL of water and aliquots of 40 μL of this cellular suspension were transferred to 1.5 ml tubes and incubated at 30 °C for 5 min for temperature equilibration. After this period, 10 μl of water containing D-glucose [0.1–40 mM] plus 0.2 μCi of ^14^C-glucose were added and the uptake was allowed for 10 seconds. After the incubation period, reaction was stopped by vigorous quenching with 1.5 ml ice-cold water. Cells were immediately harvested by vacuum filtration through nitrocellulose filters and washed two times with 1.5 ml ice-cold water. The nitrocellulose filters containing the yeast cells were transferred to 3 ml of ScintiSafeTM Econo1 scintillation liquid (Fisher Scientific), and the D-[U-^14^C] glucose taken up by cells was measured using Tri-Carb^®^ 2100TR Liquid Scintillation Counter.

## Additional Information

**How to cite this article:** Reis, T. F. *et al*. The low affinity glucose transporter HxtB is also involved in glucose signalling and metabolism in *Aspergillus nidulans. Sci. Rep.* **7**, 45073; doi: 10.1038/srep45073 (2017).

**Publisher's note:** Springer Nature remains neutral with regard to jurisdictional claims in published maps and institutional affiliations.

## Supplementary Material

Supplementary Figures and Tables

Supplementary Table S1

## Figures and Tables

**Table 1:** FetGOat analysis of genes which were significantly differently regulated in low- and high-glucose conditions (FDR = false discovery rate, BP = biological process, MF = molecular function).

GO term	Description	P-value	FDR	Ontology
**Up-regulated under low glucose concentrations**				
GO:0016054	organic acid catabolic process	0.000154	0.007646	BP
GO:0006725	cellular aromatic compound metabolic process	0.001114	0.036879	BP
GO:0046417	chorismate metabolic process	0.000576	0.023261	BP
GO:0019748	secondary metabolic process	4.68E-09	7.72E-07	BP
GO:0005975	carbohydrate metabolic process	4.49E-05	0.002871	BP
GO:0009063	cellular amino acid catabolic process	8.64E-06	0.000777	BP
GO:0043648	dicarboxylic acid metabolic process	7.73E-06	0.000765	BP
GO:0046395	carboxylic acid catabolic process	0.000154	0.007646	BP
GO:0016491	oxidoreductase activity	1.68E-07	6.14E-05	MF
GO:0051119	sugar transmembrane transporter activity	0.000312	0.045562	MF
**Down-regulated under low glucose concentrations**				
GO:0008152	metabolic process	0.001096	0.028103	BP
GO:0046365	monosaccharide catabolic process	0.001836	0.039959	BP
GO:0010467	gene expression	2.55E-08	2.97E-06	BP
GO:0071841	cellular component organization or biogenesis at cellular level	0.00096	0.026033	BP
GO:0034641	cellular nitrogen compound metabolic process	0.000842	0.024157	BP
GO:0006090	pyruvate metabolic process	0.001836	0.039959	BP
GO:0051169	nuclear transport	0.000587	0.019051	BP
GO:0006396	RNA processing	4.87E-12	8.04E-10	BP
GO:0070925	organelle assembly	0.000648	0.019982	BP
GO:0034660	ncRNA metabolic process	3.69E-16	7.32E-14	BP
GO:0006412	translation	1.61E-06	0.000114	BP
GO:0006084	acetyl-CoA metabolic process	0.002115	0.045024	BP
