# Supplementary material for: The low affinity glucose transporter HxtB is also involved in glucose signalling and metabolism in Aspergillus nidulans
Source: Sci Rep. 2017 Mar 31;7:45073. doi: 10.1038/srep45073 (PMC5374493; doi:10.1038/srep45073)
Supplement: Supplementary Figures and Tables [file srep45073-s1.pdf]

The low affinity glucose transporter HxtB is also involved in glucose signalling and metabolism in *Aspergillus nidulans.*

Thaila Fernanda dos Reis[1], Benjamin M. Nitsche[2], Pollyne Borborema Almeida de Lima[1], Leandro José de Assis[1], Laura Mellado[1], Steven D. Harris[3], Vera Meyer[2], Renato A. Corrêa dos Santos[4], Diego M. Riaño-Pachón[4], Laure Nicolas Annick Ries[1*] and Gustavo H. Goldman[1*]

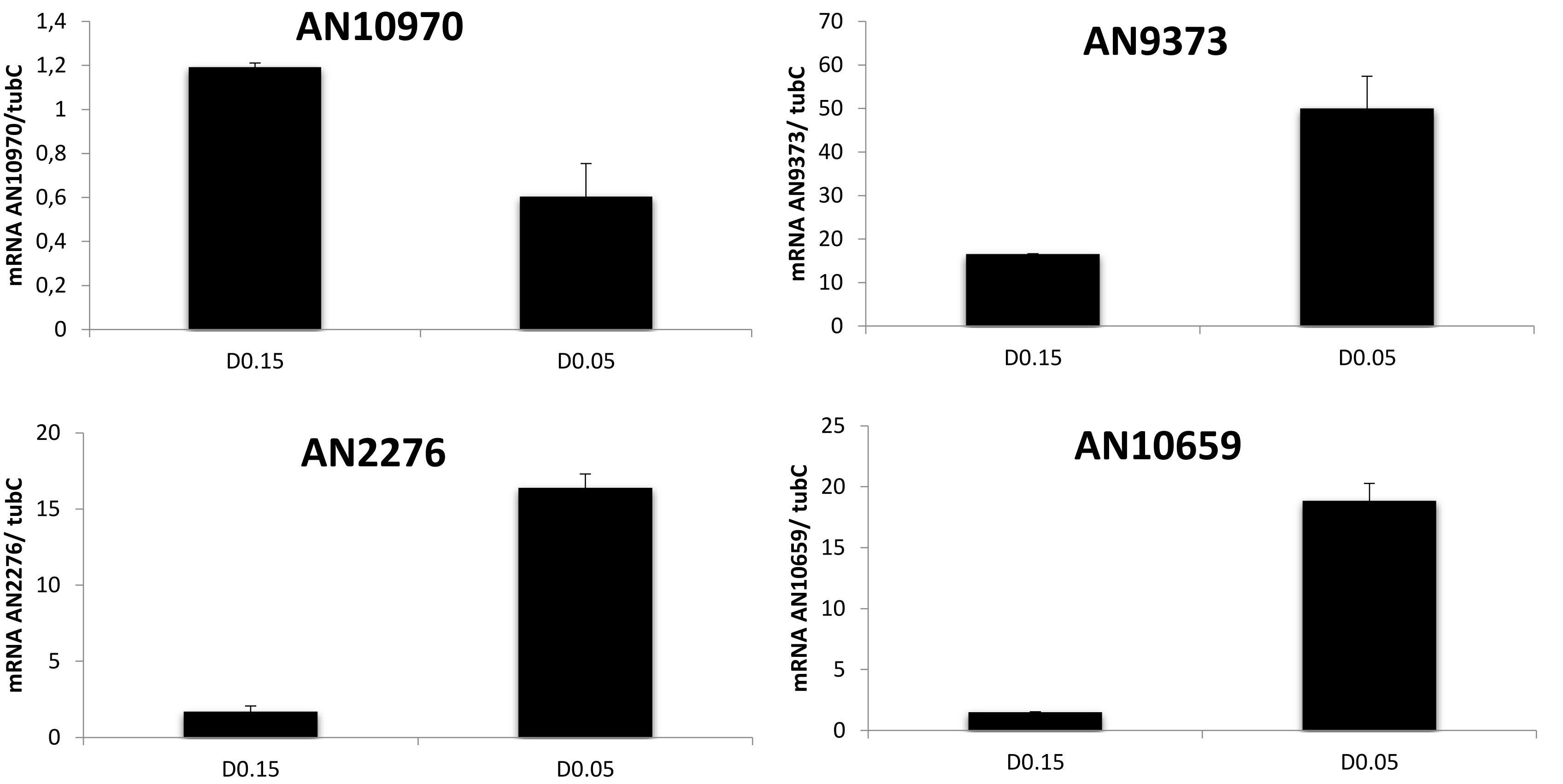

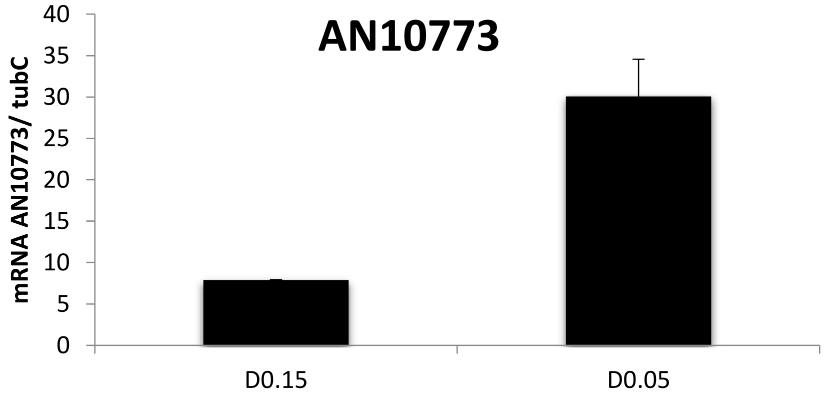

Supplementary Figure S1: Validation of RNA-seq data. Expression of five randomly chosen genes as determined by qRT-PCR under chemostat steady-state conditions. Gene expression values were normalised by the excpression of *tubC*. Standard deviations are shown for biological triplicates.

A.

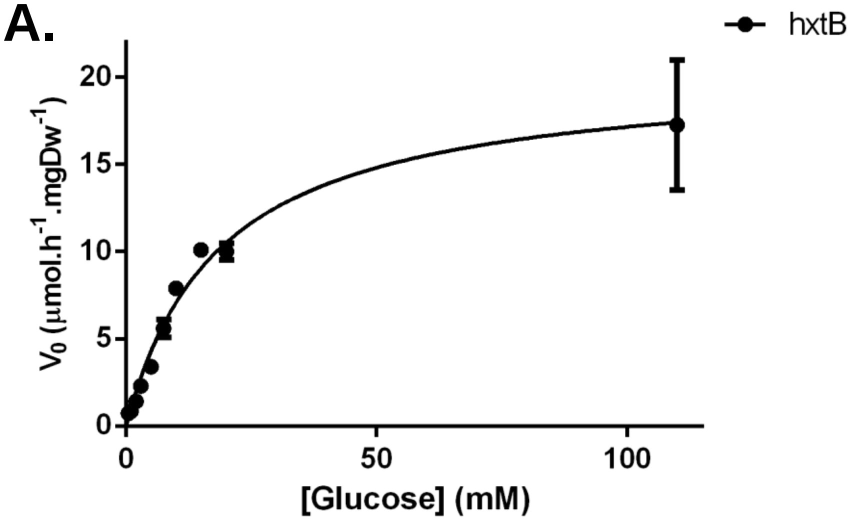

V_{max}	K_m
18.82 ± 1.52	15.54 ± 3.2

B.

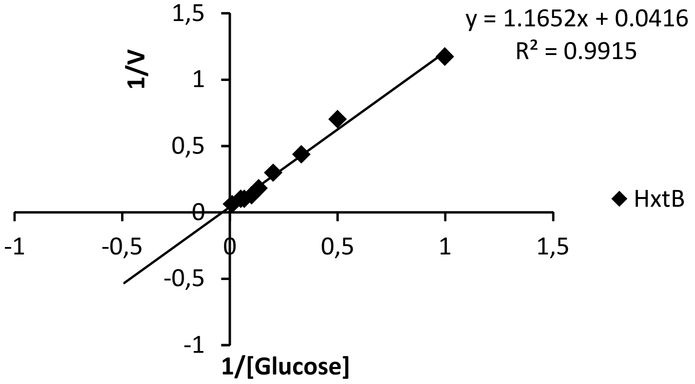

Supplementary Figure S2. HxtB is a low affinity glucose transporter. (A.) Michaelis-Menten saturation curve of HxtB in the presence of different glucose concentrations and the subsequently derived Vmax and Km values. Standard deviations are shown for three biological replicates. (B.) Lineweaver-Burk plot of the enzyme kinetics shown in (A.).

A.

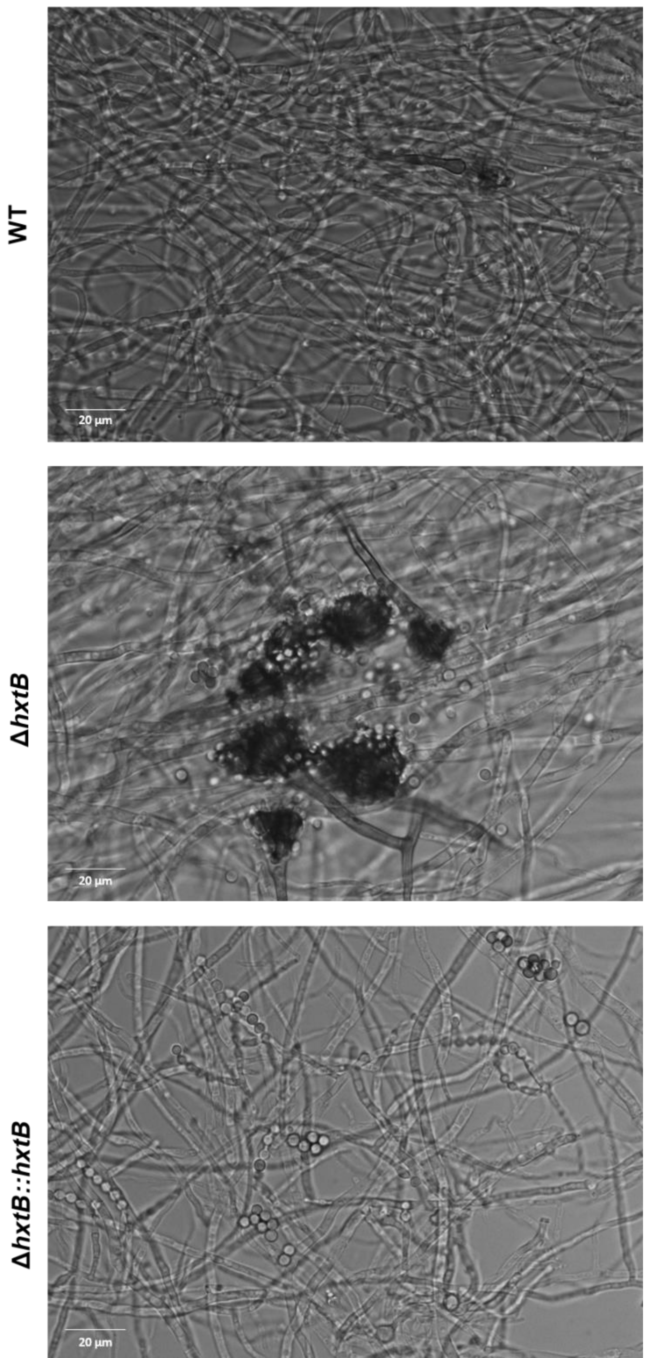

B.

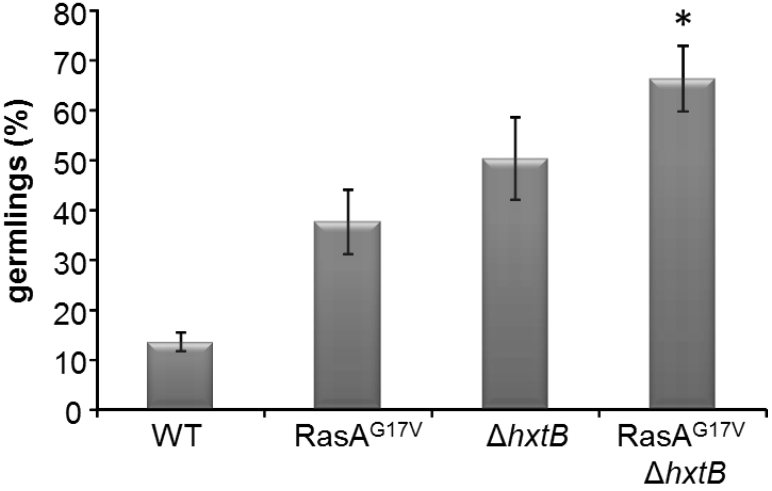

Supplementary Figure S3. HxtB is involved in fungal developmental processes. **A.** Microscopy of the wild-type (TN02a3), Δ*hxtB* and Δ*hxtB*::*hxtB* strains. Conidia were grown for 16h in minimal medium supplemented with 1% glucose and then transferred to minimal medium without carbon source for 32h. **B.** Percentage of germination, as determined by microscopy, of different strains when incubated for 16 h in minimal medium without any carbon source.

Supplementary Table S2. Strains and plasmids used in this study

Strains/plasmids	genotype	Reference
S. cerevisiae		
SC9721	MATa his 3-D200 URA 3-52 leu2D1 lys 2D202 trp 1D63	FGSC
A. nidulans		
TN02A3	pyroA4 pyrG89; chaA1; ΔnKuA::argB	(66)
A4 (Glasgow wild-type)	*veA*+	FGSC
ΔhxtB	pyroA4 pyrG89; chaA1; ΔnKuA::argB; ΔhxtB::pyroA4	(32)
hxtB::GFP TN02A3	pyrG89; pyroA4; Δnku70::argB; hxtB::GFP::pyrG	(23)
Ras^{G17V} (RKIS28.5)	*pabaA1, yA2*, *alc*A*(p)*::*ras*AG17V::*arg*B	(67)
RasG17V ΔhxtB	*pabaA1, yA2*, *alc*A*(p)*::*ras*AG17V::*arg*B; ΔhxtB::pyroA4	This study
ΔpkaA	pyrG89; wA3; argB2; ΔnkuAku70::argB pyroA4; sE15 nirA14 chaA1 fwA1; ΔpkaA::pyrG	(68)
ΔpskA	pyrG89; wA3; argB2; ΔnkuAku70::argB pyroA4; sE15 nirA14 chaA1 fwA1; ΔpskA::pyrG	(68)
pkaA::GFP	pyroA4; pyrG89; chaA1; ΔnKuA::argB; pkaA::GFP pyrG	(17)
pkaA::GFP ΔhxtB	pyroA4; pyrG89; chaA1; ΔnKuA::argB; pkaA::GFP pyrG; ΔhxtB::pyroA4	This study
Plasmids		
pRS426	ampR lacZ URA3	(69)

References

17. De Assis, L. J. *et al. Aspergillus nidulans* protein kinase A plays an important role in celullase production. *Biotechnol. Biofuels* **8,** 10.1186/s13068-015-0401-1 (2015).

23. Dos Reis, T. F., de Lima, P. B., Parachin, N. S., Mingossi, F. B., de Castro Oliveira, J. V., Ries, L. N. & Goldman, G. H. Identification and characterization of putative xylose and cellobiose transporters in *Aspergillus nidulans*. *Biotechnol Biofuels* **9,** 10.1186/s13068- 016-0611-1 (2016).

32. Dos Reis, T. F. *et al.* Identification of glucose transporters in *Aspergillus nidulans*. *PLoS One* **8,** 10.1371/journal.pone.0081412 (2013).

66. Nayak, T. A Versatile and Efficient Gene-Targeting System for *Aspergillus nidulans*. *Genetics* **172,** 1557–1566 (2005).

67. Shimizu, K., Hicks, J. K., Huang, T. P. & Keller, N. P. Pka, Ras and RGS Protein Interactions Regulate Activity of AflR, a Zn(II)2Cys6 Transcription Factor in *Aspergillus nidulans*. *Genetics* **165,** 1095–104 (2003).

68. De Souza, C. P. *et al.* Functional analysis of the *Aspergillus nidulans* kinome. *PLoS One* **3,** 58008, 10.1371/journal.pone.0058008 (2013).

69. Teepe, A. G., Loprete, D. M., He, Z., Hoggard, T. A. & Hill, T. W. The protein kinase C orthologue PkcA plays a role in cell wall integrity and polarized growth in *Aspergillus nidulans*. *Fungal Genet Biol*. **44,** 554–562 (2007)

Supplementary Table S3. Primers used in this study

Primer name	Sequence (5´- 3´)
GFP pRH195 F	GAATTAATAAAAGTGTTCGCTTAACGCCAAGCTTGCATGC
GFP pRH195 R	GGAACACGGGGAATGAGTAAAGGAGAAGAACTTTTCACTGG
pyrG F	ATTCTGTCTGAGAGGAGGCACTGATGCG
pyrG R	GAATTCGCCTCAAACAATGCTCTTCACC
Pyro F	TGGTATCATGGTTGTTGGGTC
Pyro R	AGCATCCACATGATCGACAG
tubC SYBR F	AGCTGGCGGTAACAAATACG
tubC SYBR R	ACCTGATCCACCAATTCTGC
hxtB SYBR F	AACCTGAGCGGCTATGTGAT
hxtB SYBR R	AACAGTGGTGGAGGCAGTCT
brlA SYBR F	CACTCAAAAGAGAAGCCGCA
brlA SYBR R	CCGTTTGCTATGGGTCTTCG
AN10970 SYBR F	CAAGCGGACAAAAGAACGGA
AN10970SYBR R	CTTCCTTCATCGGCACTGC
AN9373 SYBR F	CTCCCAGAACTGAGCCCTAC
AN9373 SYBR R	ATATCCAGGTTCCCTCGTCC
AN2276 SYBR F	ATGGTGATTTGGGTTGTGCT
AN2276 SYBR R	CGAGATACATATTCAGCCTCCG
AN10659 SYBR F	ATGGCATGGAGACGGAGAAA
AN10659 SYBR R	TCGCTATCATCGCTTCACCT
AN10773 SYBR F	ACTCCAGAACTCCCACACTG
AN10773 SYBR R	CCAGATCCCAGCCATCAAGA
